# Physiological Responses to a Single Low-Dose of *Bacillus anthracis* Spores in the Rabbit Model of Inhalational Anthrax

**DOI:** 10.3390/pathogens9060461

**Published:** 2020-06-11

**Authors:** Sarah C. Taft, Tonya L. Nichols, Stephanie A. Hines, Roy E. Barnewall, Gregory V. Stark, Jason E. Comer

**Affiliations:** 1 U.S. Environmental Protection Agency, National Homeland Security Research Center, Cincinnati, OH 45224, USA; Nichols.tonya@epa.gov; 2 Formerly of Battelle Memorial Institute, Columbus, OH 43201, USA; sj.hines@hotmail.com (S.A.H.); starkg72@gmail.com (G.V.S.); 3 Battelle Memorial Institute, Columbus, OH 43201, USA; barnewallr@battelle.org; 4 Institutional Office of Regulated Nonclinical Studies, University of Texas Medical Branch at Galveston, Galveston, TX 77555, USA; jscomer@utmb.edu; 5 Department of Microbiology and Immunology, University of Texas Medical Branch at Galveston, Galveston, TX 77555, USA; 6 Sealy Institute for Vaccine Sciences, University of Texas Medical Branch at Galveston, Galveston, TX 77555, USA; 7 Center for Biodefense and Emerging Infectious Diseases, University of Texas Medical Branch at Galveston, Galveston, TX 77555, USA

**Keywords:** *Bacillus anthracis*, anthrax, low-dose, dose–response, physiological response

## Abstract

Credible dose–response relationships are needed to more accurately assess the risk posed by exposure to low-level *Bacillus anthracis* contamination during or following a release. To begin to fill this knowledge gap, New Zealand White rabbits were implanted with D70-PCT telemetry transmitters and subsequently aerosol challenged with average inhaled doses of 2.86 × 10^2^ to 2.75 × 10^5^ colony forming units (CFU) of *B. anthracis* spores. Rabbits exposed to a single inhaled dose at or above 2.54 × 10^4^ CFU succumbed with dose-dependent time to death. Death was associated with increases above baseline in heart rate, respiration rate, and body temperature and all rabbits that died exhibited bacteremia at some point prior to death. Rabbits that inhaled doses of 2.06 × 10^3^ CFU or lower survived to the end of the study and showed no or minimal adverse changes in the measured physiological responses in response to the challenge. Moreover, no bacteremia nor toxemia were observed in rabbits that survived to the end of the study. Overall, the data indicate that challenge doses of *B. anthracis* below the level sufficient to establish systemic infection do not produce observable physiological responses; however, doses that triggered a response resulted in death.

## 1. Introduction

When intentionally released as a biological weapon, *Bacillus anthracis* has the potential to cause significant illness and lethality in an exposed population [1]. The release of *B. anthracis* spores poses a unique hazard due to the demonstrated lethality of inhalation anthrax and the persistence of spores after release [1,2,3]. The risk evaluation of contaminated sites requires the ability to model the inhalation hazard posed by exposure to low levels of spores re-aerosolized from surface deposits prior to or subsequent to decontamination [2]. *B. anthracis* is one of the most highly studied biothreat agents [4], yet there is no technical consensus on an appropriate dose–response relationship to describe the human health effects of exposure [5]. The scarcity of dose–response data and accompanying pathophysiological measurements are significant data gaps that currently limit progress toward determining a *B. anthracis* dose–response relationship relevant to human low-dose exposure. Data from animal models are necessary to model the human dose–response relationship because there are no environmental or dose data associated with any human cases of anthrax [5,6].

The challenge in the development of a human dose–response relationship for inhalation anthrax absent human dose or environmental data is illustrated by studies from the Sverdlovsk anthrax outbreak of 1979. The Sverdlovsk outbreak resulted from the accidental release of *B. anthracis* from a Soviet bioweapons facility and is the largest recorded human inhalation anthrax outbreak [7]. However, data are not available for release location, source strength, environmental measurement, or human exposure doses [7]. Data gaps for exposure have been addressed by the assumption of a nonhuman primate dose–response relationship where Sverdlovsk response rates could be used to identify the potential dose levels. For example, the identification of the Sverdlovsk release location and source strength considered the similarity between measured attack rates and expected attack rates that were developed using Gaussian plume modeling, an assumed human inhalation rate, and two possible dose–response relationships [7,8]. A second study reported on the relative fit of several commonly used dose–response models (e.g., probit, exponential) for inhalation anthrax relative to the Sverdlovsk data, which necessitated the assumption of a median lethality value to anchor the dose estimate across differing models [4]. Given the necessary assumption of a dose–response relationship to estimate the Sverdlovsk human exposure doses, these data cannot provide a unique data set to develop a human dose–response relationship for inhalation anthrax.

The virulence of *B. anthracis* is predicated upon the production of an anti-phagocytic capsule and the anthrax toxins [9,10]. The two toxins, lethal toxin and edema toxin, are formed through the interaction of three polypeptides, protective antigen (PA), lethal factor (LF), and edema factor (EF). Established animal models for human inhalation anthrax include the rabbit and nonhuman primate [11,12,13]. Commonalities in both inhalation anthrax pathology and outcomes are described among the rabbit, nonhuman primate, and human [12,14,15,16,17,18]. In the rabbit, Zaucha et al. [18] identified pathologically significant findings in the spleen, lymph nodes, gastrointestinal tract, and adrenal glands. Lesions were also noted in the mediastinum, brain, bone marrow, kidney, thymus, heart, and ovaries [18].

Given the lack of applicable data from studies using high-dose challenges, studies inclusive of low-doses are necessary to address known data gaps. Low-dose challenge data will contribute to the modeling of anthrax pathophysiology in a more realistic dose range relative to potential human exposure and assist in the development of predictive models for human health risk assessment, including dose–response modeling and low-dose extrapolation. This paper reports on a study performed in the rabbit model to evaluate physiological responses following a single inhaled dose of *B. anthracis* Ames strain spores for a range of low to high doses. To date, the survival data have been used to model responses to single exposures of *B. anthracis* spores [19], adding to the work done by Gutting et al. [20] on dose–response modeling using the rabbit model. Here we present the study design, data sets, and initial data analysis to increase access by the broader research community to the study data.

## 2. Materials and Methods

All of the following select agent work was conducted at the Battelle Biomedical Research Center (West Jefferson, OH, USA) in a Biosafety Level-3/Animal Biosafety Level-3 laboratory registered with the U.S. Centers for Disease Control and Prevention (CDC) and inspected by the U.S. Department of Defense and the U.S. Department of Agriculture. The animal procedures and protocols used were pre-approved by Battelle’s Institutional Animal Care and Use Committee (IACUC) and the Department of Defense’s Animal Care and Use Review Office (ACURO). The research was conducted in compliance with the federal Animal Welfare Act and followed the principles in the Guide for the Care and Use of Laboratory Animals from the National Research Council [21]. The institution where the research was conducted is fully accredited by the Association for the Assessment and Accreditation of Laboratory Animal Care International (AAALAC).

All work was conducted following an approved Test/Quality Assurance Plan to ensure that sufficient quality objectives and performance criteria were met for determination of the adequacy of data generated during the study. Further detail on the quality assurance process can be found in the project report [22].

### 2.1. Bacillus anthracis Ames Spores and Aerosol Challenge

The *B. anthracis* Ames strain spores were prepared and characterized as previously described in Barnewall et al. [2]. Following growth in a fermentor, cultures exhibiting >80% refractile spores, as determined by phase-contrast microscopy, were centrifuged at approximately 10,000–12,000× *g* for 15–20 min at 2–8 °C. The resultant pellet was washed twice and resuspended in ice-cold, sterile water. The suspension was heat-shocked by incubating at 60 °C for 45–60 min to kill vegetative cells, and centrifuged and washed a minimum of two times in ice-cold, sterile water to remove cellular debris. The spore preparation was purified by centrifuging the sample through a gradient ice-cold, sterile 58% Hypaque-76 (Nycomed Amersham, Princeton, NJ, USA) at 9000× *g* for 2 h at 2–8 °C. The resultant pellet was washed and resuspended in ice-cold, sterile water and evaluated by phase-contrast microscopy. Spores used in the negative control group were inactivated with 4.0 × 10^6^ rad doses of gamma irradiation in a Centers for Disease Prevention and Control (CDC) laboratory using a method described by Dauphin et al. [23]. The CDC verified inactivation using plating of samples. All rabbits were challenged with aerosolized *B. anthracis* Ames spores as previously described [2]. Briefly, rabbits were exposed to a muzzle-only aerosols of *B. anthracis* Ames strain spores generated by a 3-jet Collison nebulizer (BGI, Waltham, MA, USA). Whole body plethysmography was used to determine the accumulated tidal volume necessary to achieve the desired dose. The concentration of spores in the aerosol was determined by sampling of the atmosphere with an impinger (Model 7541, Ace Glass Inc.) and subsequent quantitative plating on blood agar. The targeted doses were log increases from 1.0 × 10^2^ to 1.0 × 10^5^ and a high dose of 1.05 × 10^7^. As described in Barnewall et al. [2], the actual inhaled doses were calculated using the product of aerosol concentration and total inhaled tidal volume determined via real-time plethysmography.

Thirty-five male pathogen-free NZW rabbits (*Oryctolagus cuniculus*) weighing approximately 3.5 kilograms (kg) were purchased from Covance (Denver, PA, USA). The rabbits were fitted with vascular access ports (VAPs) and a Data Sciences International (St. Paul, MN, USA) model D70-PCT telemetric device prior the start of the study. The rabbits were randomized by weight into six groups of five rabbits per group. The rabbits within each group were randomized for challenge order based on ear tag numbers provided by the supplier. The SAS^®^ software PLAN procedure (SAS Institute, Inc., Cary, NC, USA) was used to randomize the animals. The rabbits were challenged according to randomization order and challenge dose group. Survival and telemetry data were collected continuously throughout the study for 21 days after the challenge day.

### 2.2. Necropsy and Histopathology

Gross necropsy was performed on all animals. Lungs and gross lesions (including brain, bronchial and mediastinal lymph nodes, thymus, small intestine, and skin) were collected and examined by histology to aid in the confirmation that death was due to *B. anthracis* infection. The collected tissues were placed in 10% neutral buffered formalin, processed to approximately 5 µm slides, stained with hematoxylin and eosin, and examined histologically by a board-certified pathologist. All microscopic findings were graded semi-quantitatively according to the following scale, with the associated numerical score used to calculate average severity grades for each lesion by group:

- Minimal (Grade 1): the least detectible lesion (i.e., <5%);
- Mild (Grade 2): an easily discernible lesion (i.e., 5% to <25%);
- Moderate (Grade 3): a change affecting a large area of the represented tissue (i.e., 25% to 50%);
- Marked (Grade 4): a lesion that approached maximal (i.e., >50%).

Gross and microscopic diagnoses were entered into the PATH/TOX SYSTEM (Xybion Medical Systems Corporation, Cedar Knolls, NJ, USA) for data tabulation and analysis.

### 2.3. Telemetry Analysis

Body temperature, electrocardiogram activity, and cardiovascular function (heart rate and respiratory pressure) were monitored for 30 s every 15 min for seven days pre-challenge (baseline) through the end of the study. Each animal’s cage was equipped with a Data Sciences International telemetry receiver. The transmitters, receivers, consolidation matrices, cabling, and computers using the Dataquest A.R.T.^™^ data acquisition and analysis software were all components of the PhysioTel^®^ Telemetry System (Data Sciences International). The Dataquest A.R.T.^™^ telemetry software collected the telemetry parameters mentioned above. The process to determine departure from baseline in telemetric data for an individual animal is described in the Statistical Analysis section.

### 2.4. Hematology and Clinical Chemistry Analysis

Blood samples were collected via the VAPs on study days −3, 1, 2, 3, 7, and 14. If a VAP failed, the animal was sedated with acepromazine, and blood was collected via the medial auricular artery or the marginal ear vein. Terminal samples were collected from animals that succumbed to disease if the blood had not clotted by the time of collection. Complete hematological analysis was performed using the Advia™ 120 Hematology Analyzer (Siemens Healthcare Diagnostics, Deerfield, IL, USA). Plasma was assayed via the Advia® 1200 chemistry analyzer (Siemens Healthcare Diagnostics).

### 2.5. Bacteremia Analysis

For qualitative bacteremia culture analysis, whole blood was streaked over blood agar plates (Hardy Diagnostics, Santa Maria, CA, USA) and observed to detect growth and morphology consistent with *B. anthracis* after a minimum incubation of 48 h at 37 °C ± 2 °C. Quantitative counts were achieved by 10-fold serial dilutions of the blood samples in Dulbecco’s phosphate buffered saline (Hardy Diagnostics) from 1 × 10^−1^ to 1 × 10^−9^ and spread plating 100 microliters (µL) of each dilution onto tryptic soy agar (Hardy Diagnostics) in triplicate. The limit of detection (LOD) and lower limit of quantification (LLOQ) for detecting bacteremia were 100 CFU/mL and 2500 CFU/mL, respectively.

### 2.6. Toxemia Analysis

The circulating PA ELISA was performed as described in Comer et al. [24]. The LOD for this assay was 2.0 ng/mL and the LLOQ was 4.9 ng/mL.

### 2.7. Anti-PA IgG Analysis

Microtiter plates were coated with purified rPA. Unknown test samples, anti-PA IgG reference standard serum, and positive control sera are added to the microtiter plate. The PA-specific antibodies present in the samples/standards were allowed to bind to the rPA coated on the plate. After washing, the bound anti-PA antibodies were then detected by a species-specific anti-gamma chain IgG–HRP conjugate followed by addition of a peroxidase substrate. The optical density (OD) values for each plate were then read on a microplate reader (ELx800; BioTeK, Winooski, VT, USA) at a wavelength of 405 nanometers using a 490 nanometer reference wavelength. Results were reported in μg/mL of anti-PA IgG for each unknown test sample.

### 2.8. Statistical Analysis

Estimates and exact Clopper–Pearson 95% binomial confidence intervals for the proportion of surviving animals were calculated for each group. An overall two-sided Fisher’s exact test was performed to determine if the proportions of surviving animals were significantly different among the groups. If the overall Fisher’s exact test was significant, then pairwise two-sided Fisher’s exact tests were performed to determine which pairs of groups were significantly different from each other. A Bonferroni–Holm adjustment was made to maintain an overall 0.05 level of significance for the multiple pairwise comparisons. A logistic regression model was fitted to the survival data as a function of the base 10 logarithm of the estimated inhaled dose to determine the effect of dose on lethality. The LD_50_ was estimated from the logistic regression model, along with 95 percent Fieller’s confidence intervals.

The mean telemetry value was computed for every 15 min clock time reported in military time units (0000, 0015,…, 2400) at baseline. The average for each hour of the day was calculated across the baseline days. These averages were then subtracted from the time-matched values before and after challenge. Each observation was then baseline adjusted according to the associated clock time and six-hour averages were computed from the baseline adjusted values using the following intervals: 2400–0600 (inclusive), 0600–1200 (inclusive), 1200–1800 (inclusive), and 1800–2400 (inclusive). The baseline adjusted data before challenge were used to set abnormality thresholds for each parameter.

In order to determine if the baseline adjusted telemetry results were significantly different among the groups, analysis of variance (ANOVA) models were fit to the baseline adjusted six-hour average telemetry values with an effect for group at each study time. Least square mean estimates from the ANOVA models were calculated and approximate *t*-tests were performed to determine whether, for each group, there was a significant shift in the telemetry values between baseline and each study time, after adjusting for the clock time. Additionally, Tukey’s multiple comparisons procedure was performed to determine which pairs of groups had mean baseline adjusted telemetry values that were significantly different than each other. Under the Tukey procedure, the set of all comparisons within each parameter and study time combination are made at a joint 95% confidence level.

The standard deviation of each 6-hour average at baseline was calculated and used to form the upper and lower limits for indications of abnormality for telemetry measures of heart rate, respiration rate, and body temperature. The upper limit was defined to be three standard deviations above zero, while the lower limit was defined to be three standard deviations below zero. An animal was found to be abnormal if two consecutive baseline-adjusted 6-hour averages were outside the upper or lower limits following challenge. The time of onset for abnormality was defined as the time associated with the second abnormal value during the first occurrence of two consecutive abnormal values following challenge. The end of abnormality was defined as the time associated with the last abnormal value during the last occurrence of two consecutive abnormal values following challenge. Therefore, the duration of abnormality was defined as the difference between the time associated with the end of abnormality and the time associated with the onset of abnormality. Fisher’s exact tests were performed to test for significant differences in the proportion of animals abnormal among the groups. Log-rank tests were performed to test for significant differences in time to abnormality and duration of abnormality among the groups.

The PA and quantitative bacteremia data were log-transformed for the statistical analyses. The assumption of normality was deemed reasonable for the log-transformed values. All PA measurements less than the LOD were replaced with one-half of the LOD. All quantitative bacteremia measurements less than the LOD (reported as zero CFU/mL) were replaced with one-half of the LOD. Furthermore, if an observation was positive for *B. anthracis* but less than the LOQ, then it was replaced with one-half of the LOQ. The replacement of measurements less than the LOD or LOQ with one-half of its value was conducted only for the statistical analysis; reported values unrelated to the statistical analysis were not altered. T-tests were performed to determine if the geometric mean was significantly greater than the LOD for each parameter, group, and study day. For each hematology and clinical chemistry parameter, ANOVA and Tukey’s multiple comparisons procedures were performed at each study time to determine if the mean shifts from baseline were significantly different among the groups. The ANOVA models were also used to test if parameter values were significantly different from baseline for each group and time point.

Statistical analyses were conducted using SAS^®^. Details on the statistical tests conducted can be found in documentation for the software.

## 3. Results

### 3.1. Mortality

**Figure 1:**
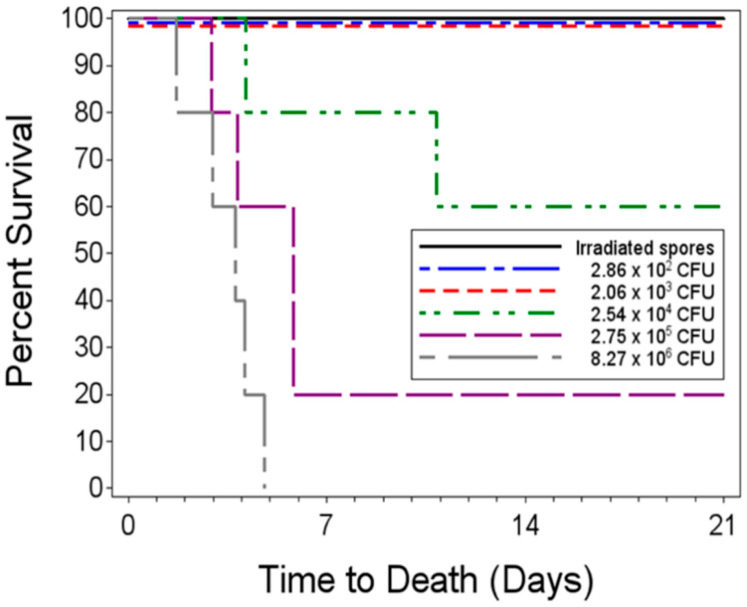
Kaplan–Meier curves representing time to death from the challenge day (Day 0) and survival data for each single low-dose group. Five groups of five rabbits were challenged with 2.86 × 10^2^ (±4.32 × 10^1^) CFU to 8.27 × 10^6^ (1.69 × 10^6^) CFU. As a negative control, one group of five rabbits was exposed to 1.05 × 10^7^ of gamma-irradiated spores. The rabbits were monitored for 21 post-challenge for mortality.

To determine the dose–response relationship for a single low-dose aerosol exposure to live *B. anthracis* spores, five groups of five rabbits were aerosol challenged and received inhaled doses ranging from 2.86 × 10^2^–8.27 × 10^6^ CFU from a spore suspension. The groups of challenged animals will be referred to by the mean inhaled dose for each group. The negative control group was challenged with irradiated spores. All rabbits in the negative control group and those challenged with less than an average of 2.06 ×10^3^ CFU lived to the end of the study. Two of the five rabbits in the group challenged with a mean of 2.54 × 10^4^ CFU died 4.14 and 10.85 days post-challenge, respectively. Four of the five rabbits challenged with 2.75 × 10^5^ CFU died, with a mean time to death of 4.64 days post-challenge. All five of the animals challenged with 8.27 × 10^6^ CFU succumbed to infection, with a mean time to death of 3.47 ± 1.18 days post-challenge (Figure 1). The number of inhaled spores for each rabbit are listed in Supplementary Table S1.

Based on the unadjusted Fisher’s exact tests, the proportions of surviving animals in the irradiated spore group and in the two lowest dose groups (2.8 × 10^2^ CFU and 2.06 × 10^3^ CFU) were significantly greater than those in the 2.75 × 10^5^ and 8.27 × 10^6^ CFU dose groups. Additionally, the 2.54 × 10^4^ CFU dose group showed significantly greater times to death than the highest dose group. However, after adjusting for the multiple comparisons, there were no significant pairwise differences between any of the groups of exposed animals since the multiple comparisons have a more stringent requirement for significance.

The logistic regression model fitted to the survival data indicated a significant dose–response relationship with increased inhaled doses being associated with decreased probabilities of survival, as evidenced by the significant *p*-value associated with the estimated slope coefficient of −2.21 (*p* = 0.0147). The estimated LD_50_ was 5.18 × 10^4^ CFU with a 95% Fieller confidence interval ranging from 6.14 × 10^3^ CFU to 7.27 × 10^5^ CFU.

### 3.2. Telemetry

**Figure 2:**
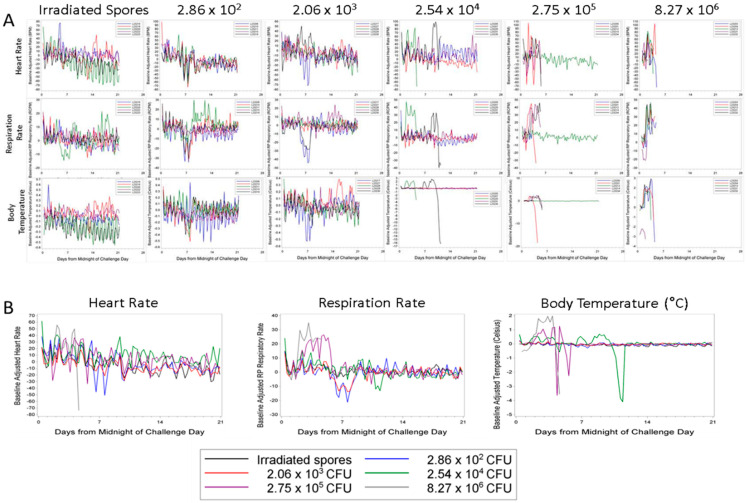
Telemetric monitoring of physiological responses to low-dose exposure. The rabbits were surgically implanted with telemetry units (D70-PCT transmitters, Data Sciences International) prior to being placed on study. Each D70-PCT transmitter contained one pressure lead and one biopotential lead. Body temperature, electrocardiogram activity, and cardiovascular function were monitored for 30 s every 15 min for seven days pre-challenge (baseline) through the end of the study. (**A**) shows individual rabbit data (each line is an individual rabbit). (**B**) shows group mean data (each line represents the group mean).

Individual animal baseline adjusted telemetry data are illustrated in Figure 2A; group means are shown in Figure 2B. There was not a clear correlation between increased heart rate and death, as several of the rabbits experienced tachycardia and lived to the end of the study. However, all rabbits that died in the study except L23200 (2.75 × 10^5^) were tachycardic just prior to death (Figure 2A). By study day 1 at 0600–1200, all groups had experienced significant increases in the group mean heart rate compared to baseline. The elevated heart rates continued until study day 4 for all exposure groups, except for those rabbits that received a mean dose of 2.54 × 10^4^ CFU. The group mean heart rate of 2.54 × 10^4^ CFU group continued to exhibit some statistically significant increases from baseline through study day 11 (Figure 2B). There was no definitive explanation for the differences in the length of time the animals experienced significant increases in heart rate; they were housed in the same room and exposed to the same environmental stimuli.

Each rabbit that succumbed to disease showed a pronounced increase in respiration rate (greater than 30 respiratory cycles per minute above baseline) (Figure 2A). When comparing the group mean changes, there were significant increases from baseline in the 2.75 × 10^5^ CFU challenge group from study day 2 at 0600–1200 through study day 5 at 1200–1800 and in the 8.27 × 10^6^ CFU challenge group from study day 2 at 0000–0600 through study day 4 at 1200–1800. These increases ranged from 20 to 30 breaths per minute over baseline values. The mean increase from baseline in these two groups was often significantly different than the mean change from baseline in at least one of the other groups during these study times (*p* < 0.05 Tukey’s test) (Figure 2B).

Similarly to the respiration rate data, increases in body temperature above baseline were dose-dependent. Rabbits that developed fulminant disease presented with an increase in body temperature of 2 to 3 °C above baseline (Figure 2A). For seven consecutive six-hour intervals beginning on study day 2 at 0600–1200, there were significant increases from baseline in the group exposed to 8.27 × 10^6^ CFU. This group showed significant increases in baseline compared to the other groups through study day 4 at 1200–1800 (*p* < 0.05, Tukey’s test). The mean increase in body temperature reached close to 2 °C above the baseline (Figure 2B). The 2.75 × 10^5^ CFU dose group also showed significant increases over baseline compared to the lower dose groups on study day 5 at 0600–1200 and 1200–1800 (*p* < 0.05, Tukey’s test). While not statistically significant from the negative controls, there were increases in mean body temperature in the 2.54 × 10^4^ CFU dose group. These increases were attributable to the two animals that died in this group on study days 4 and 10 (Figure 2A). Rabbit L23221 (8.27 × 10^6^ CFU group) showed a decrease in body temperature starting at midnight of the challenge day when compared to baseline. The cause of this drop in body temperature is not known. The animal may have had an elevated temperature prior to challenge or there may have been a problem with the transponder. However, this animal still followed the group trend and showed a spike in body temperature prior to death (Figure 2A).

In terms of the proportion of animals that exhibited abnormal telemetry parameters during the study, there were no significant differences between the groups for any parameter. In terms of time to abnormality, there were significant differences between the groups for respiratory rate and temperature. For each of these telemetry parameters, the time to abnormality in at least one of the five lower dose groups was significantly greater than that in the highest dose control group prior to the adjustments for multiple comparisons. The duration of abnormality was found to be significantly different between the groups for heart rate and respiratory rate; however, there were no significant pairwise differences between the groups for any parameter after the adjustment for multiple comparisons.

The onset of tachycardia, tachypnea, and pyrexia proceeded death by approximately 48 to 72 h in the 2.54 × 10^4^ CFU dose group. For example, rabbit L23225 died on study day 4 with the physiological challenges starting on study day 2. Rabbit L23235 of the same dose group lived until study day 11, with changes in heart and respiration rates starting on study day 9 and the increase in body temperature starting on study day 7. Three of the four non-survivors (L23201, L23214, and L23234) in the 2.75 × 10^5^ CFU dose group presented with increases in the telemetric parameters prior to death. Interestingly, rabbit L23200, of the 2.75 × 10^5^ CFU dose group, did not develop tachycardia or pyrexia prior to death on study day 3, but did have an elevated heart rate immediately prior to death. The animals in the high dose group (8.27 × 10^6^ CFU) all presented with telemetry changes, though the time of onset of change to death was 24 to 72 h.

### 3.3. Hematology and Clinical Chemistry

While heterophils were the only parameter that exhibited a dose–response relationship, significant changes from baseline were observed in platelet (PLT), WBC counts, and the numbers of circulating heterophils, lymphocytes, and monocytes. Individual hematology data and group means and standard deviations are provided in Supplementary Table S2.

**Figure 3:**
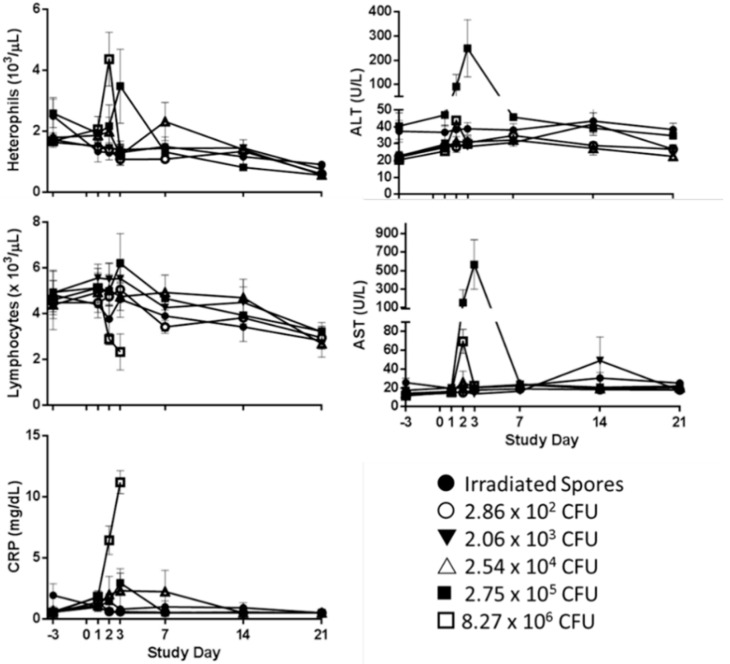
Changes in hematology and serum chemistry following low-dose exposure. Whole blood was analyzed for hematological changes with the Advia^®^ 120 Hematology Analyzer and serum chemistry was monitored with the Advia^®^ 1200 Chemistry Analyzer. The mean values for each group are represented by a different symbol. The bars indicate standard error.

The 8.27 × 10^6^ CFU dose group showed the characteristic inversion of the heterophil: lymphocyte ratio [24,25] on Day 2. This group had a significant increase in heterophils from baseline to study day 2, with a mean of 4.37 (±1.53) × 10^3^ /μL compared to 1.68 (±0.27) × 10^3^ /μL at baseline (*p* < 0.05, Tukey’s Test) (Figure 3). This shift from baseline in this group was statistically different from all other groups on study day 2 (*p* < 0.05, Tukey’s Test). While this shift from baseline was significant, only two animals had levels outside of the normal range of 0.80 to 2.90 × 10^3^ /μL [26]. On study day 3, the heterophil count peaked in the 2.75 × 10^5^ CFU dose group. Two of the three rabbits alive at this time point had heterophil levels of 4.86 × 10^3^ /μL and 4.53 × 10^3^ /μL.

C-Reactive Protein (CRP) is an indicator of stress and non-specific inflammation. CRP can also be used as a marker for liver damage. Individual rabbit clinical chemistry results and group means and standard deviations are provided in Supplementary Table S3. All groups had at least one animal with detectable levels of CRP through study day 3. There were significant increases in CRP in the two highest dose groups (2.75 × 10^5^ CFU and 8.27 × 10^6^ CFU) at study day 3 (*p* < 0.05, Tukey’s Test) with the 8.26 × 10^6^ CFU group 3.8 times higher than baseline (Figure 3). There was also a significant increase in AST in the 2.75 × 10^5^ CFU and the 8.27 × 10^6^ CFU dose groups on study day 2, and in the 2.75 × 10^5^ CFU dose group at study day 3 (Figure 3). At study day 3, the increase as a proportion of baseline in the 2.75 × 10^5^ CFU dose group showed significantly increased change in ALT, LDH, and SDH when compared to all other groups. Figure 3 shows the levels of AST and ALT for all dose groups.

### 3.4. Bacteremia, Toxemia, and Anti-PA IgG

**Figure 4:**
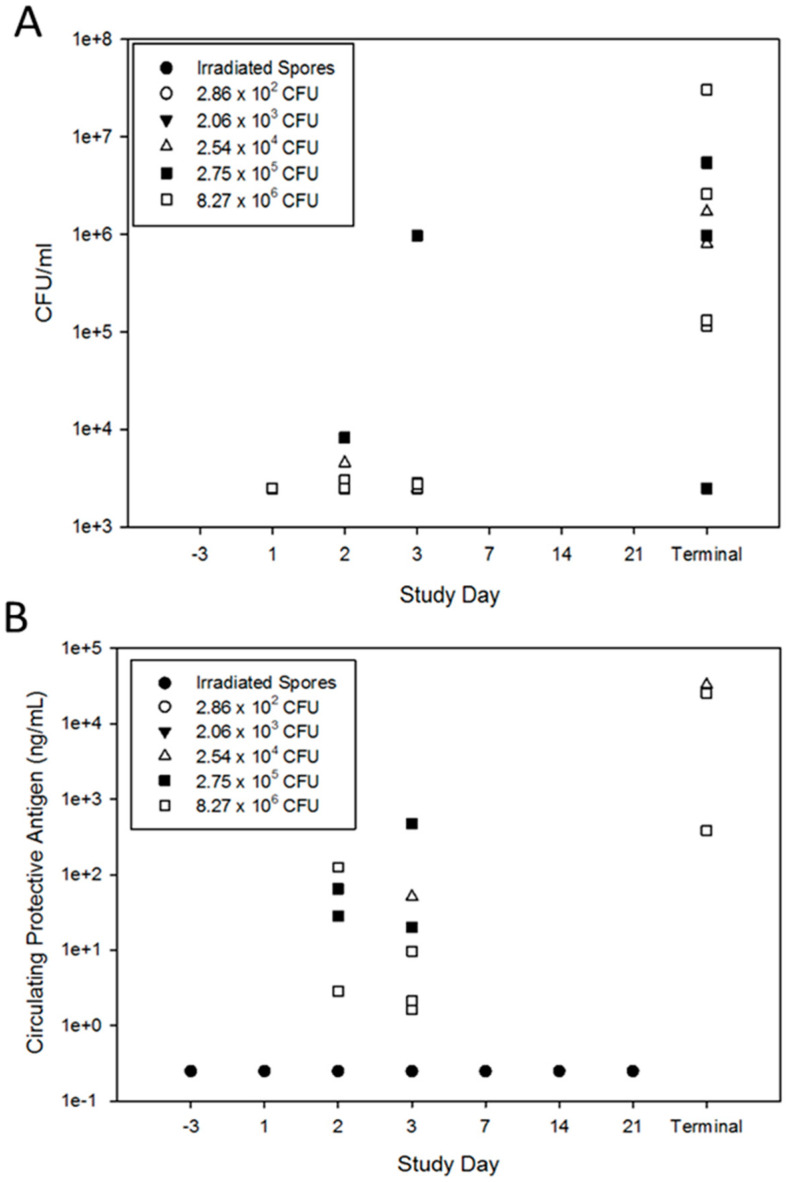
Bacteremia (**A**) and PA (**B**) levels after low-dose exposure to *B. anthracis*. Blood samples were quantitatively plated on tryptic soy agar and serum samples were assayed for PA levels via ELISA. Each dose group is represented by a different symbol. Each symbol on the graph represents an individual animal on the identified study day or the day of the animal’s death for the study day identified as terminal. The circulating levels of PA were below the limit of detection in all rabbits exposed to irradiated spores.

Figure 4A,B illustrates the bacteremia and toxemia levels after exposure to a low-dose of *B. anthracis* spores for all animals with positive bacteremia results (i.e., quantitative result or positive detection greater than LLOQ). Rabbits challenged with 2.06 × 10^3^ CFU or lower were negative for *B. anthracis* bacteremia by culture and PA in circulation by ELISA on all study days.

Two of five rabbits in the 2.54 × 10^4^ CFU dose group were bacteremic at least once during the study, and both succumbed to disease. One rabbit (L23225) had a bacterial burden of 4.53 × 10^3^ CFU/mL of blood on study day 2 and 8.03 × 10^5^ CFU/mL at the time of death (study day 4). This animal was also positive on study day 3, but the bacterial load was below the LLOQ (2.5 × 10^3^ CFU/mL). Rabbit L23225 had 51 ng/mL of circulating PA on study day 3 and this increased to 33,000 ng/mL (>ULOQ) at the time of death on study day 4 (Figure 4B). The other non-survivor (L23235) from this group had a bacterial burden of 1.70 × 10^6^ CFU/mL at the time of death (study day 11); all other samples for this animal were negative. It is not known if rabbit L23235 was toxemic as a terminal serum sample was not able to be obtained.

The lone surviving rabbit (rabbit L23212) from the 2.75 × 10^5^ dose group did not have a positive blood culture or detectable levels of PA. *B. anthracis* was cultured from the terminal samples from three of the four animals that died in this group. The fourth rabbit (L23214) that was negative for bacteremia at the time of death (5.8 days post-challenge), but was positive for bacteremia (8.23 × 10^3^ CFU/mL) on study day 2. Histology on rabbit L23214 revealed bacilli and mild suppurative inflammation in the lungs suggesting the animal died from the *B. anthracis* challenge. All but one of the rabbits that succumbed to disease in this group had detectable levels of PA at some point in the study. Rabbit L23200 did not have detectable levels PA and also had levels of bacteria below the level of quantitation. The low bacteria level and absence of PA correspond with the lack of telemetry changes in this animal. Plural effusion, characteristic of inhalation anthrax [18], was observed upon necropsy, and suppurative inflammation was noted in the lung during histological evaluation, together confirming *B. anthracis* infection as the cause of death.

All of the 8.27 × 10^6^ CFU dose group rabbits had positive bacteremia cultures at some time during the study. Only one animal (L23232) did not have positive bacteremia culture at the time of death (study day 4), but had a bacterial burden of 2.77 × 10^3^ CFU/mL of blood on study day 3. Toxemia could not be confirmed in one rabbit, L23221, in this group. This rabbit was below the LOD on Day 1 and died on Day 2 with a terminal bacteremia level of 2.58 × 10^6^ CFU/mL but a serum sample was not able to be obtained to determine PA levels.

No rabbits seroconverted during the study, suggesting that survivors were not colonized and that those that succumbed to disease died prior to initiating an IgG response. All ELISA samples had 0 μg/mL of anti-PA IgG except for one animal. Rabbit L23229 (2.06 × 10^3^ CFU) had levels of 0.386, 0.502, and 0.404 μg/mL on study days 7, 14, and 21, respectively. While these samples did produce results, they were well under the LOQ of 5.0 μg/mL and even the LOD of 1.0 μg/mL.

### 3.5. Necropsy and Histopathology

All rabbits were necropsied when they succumbed to infection or at the end of the study. Pathology findings for individual rabbits are provided in the Supplementary Table S4. Gross lesions in rabbits that succumbed to challenge with *B. anthracis* included discoloration of the brain (meninges), crusting of the skin, abdominal and thoracic cavity fluid, bronchial and mediastinal lymph node enlargement, small intestinal fluid, and thymic fluid. These gross lesions were typical of anthrax in rabbits [18] and correlated histologically with hemorrhage, necrosis, edema, and suppurative inflammation. Lungs and gross lesions were examined microscopically for evidence of anthrax. Microscopic lesions typical of anthrax as described in Zaucha et al. [18] were present in all rabbits that died on study and included minimal to moderate suppurative inflammation (predominately degenerate and viable heterophils (polymorphonuclear cells)), necrosis, hemorrhage, fibrin and/or large rod-shaped bacteria in the lungs, bronchial and mediastinal lymph nodes, skin, small intestine, brain (meninges), and thymus. While there are several studies that report pathology in the nonhuman primate for low-dose inhalation exposure (i.e., less than 10^5^ inhaled CFU/animal) to live *B. anthracis* spores [16,17,27], this is the first published study to report pathology in the rabbit after low-dose aerosol inhalation challenge that resulted in lethal inhalation anthrax.

Most rabbits challenged with live spores exhibited minimal inflammation (suppurtative or nonsuppurative) and minimal to moderate multi-nucleated giant cells in the lung tissue. The multinucleated giant cells often surrounded or contained birefringent foreign debris. These cells and debris were also seen in the lungs of two control animals, but occurred with greater frequency and severity in live spore challenged rabbits. Cell aggregates of this type were not described in Zaucha et al.’s [18] single dose study and are probably not disease-specific.

Other anthrax-related lesions were found in the lymph nodes, meninges, skin, thymus, and small intestine. Lymph node findings included hemorrhage, lymphoid necrosis, fibrin, and bacteria. There were minimal to mild intravascular bacteria, suppurative inflammation, and hemorrhaging (with vascular necrosis) primarily in the meninges of the brain of one rabbit (L23232, 8.27 × 10^6^ CFU dose group).

## 4. Discussion

The anthrax letter attacks of 2001 resulted in 12 cases of cutaneous and 11 cases of inhalational anthrax; five of the 11 individuals died from the inhalational form of the disease [28]. The processing of these spore-containing letters through the U.S. mail resulted in the contamination of numerous government and private facilities for which remediation was required [29]. As part of the remediation process, a standard of nondetection of culturable spores was used to confirm that the decontamination of buildings was successful. Over fifteen years later, the criterion for remediation remains nondetection combined with additional measures of decontamination efficacy (i.e., lines of evidence) [3]. One contributing reason for the lack of a risk-based standard is the unavailability of credible dose–response data for *B. anthracis* that could be used to estimate the risk posed by low-dose inhalation exposures. This study was conducted to develop dose–response data for a variety of endpoints for low and high-dose inhalation challenges of *B. anthracis* Ames spores in the rabbit. The primary purpose was to begin to fill known single low-dose data gaps for *B. anthracis* exposure to facilitate physiological and dose–response modeling for the rabbit and human.

All animals that received inhaled doses of 2.06 × 10^3^ CFU or less survived to the end of the study and were not observed to be bacteremic or toxemic at any time. Rabbit L23227 (2.06 × 10^3^ CFU dose group) was noted as having bacilli in the lung upon microscopic examination; however, it cannot be confirmed that these were *B. anthracis*. The entire lung was fixed in 10% buffered formalin, so the tissue could not be cultured. There may have been spore germination in the lung, as a systemic infection was not established as evidenced by measurable bacteremia. However, hematogenous spread back to the lungs cannot be ruled out, as a transient bacteria may have been missed in the blood collection schedule. Several non-surviving animals in the higher dose groups also had bacilli in the lungs. While it is widely regarded that inhaled spores are taken up by phagocytes [30,31] where they germinate [32,33] and are transported to the mediastinal lymph nodes, germination in the lungs of mice has been demonstrated [34]. However, there are several other reports that indicate no significant germination occurs in the lungs of mice challenged with *B. anthraces* spores, making hematogenous spread back to the lungs most likely scenario for rabbit L23227.

Death was associated with tachycardia, tachypnea, pyrexia, and heterophilia. The animals that died from infection exhibited pathology findings consistent with inhalational anthrax. Enzymes indicative of liver damage were also elevated; however, there was no gross evidence of liver damage. These observations correspond to the findings of Lawrence et al. [35], who evaluated the physiological responses of Dutch belted rabbits exposed to 1.00 × 10^7^ *B. anthracis* spores via nasal instillation. The Dutch belted rabbits also presented with tachycardia (defined by heart rate greater than 300 bpm), fever (not defined), and a statistically significant increase in heterophils at some point in time prior to death [35]. This breed of rabbit also showed significant increases in ALT levels. Interestingly, no significant increase in AST was observed in the Dutch Belted rabbits, whereas there was a significant increase in the NZW rabbits presented here. This may be the result of differences in the two breeds of rabbit used in the studies.

The only survivor in the 2.75 × 10^5^ CFU dose group (L23212) received an actual inhaled dose of 3.29 × 10^5^ CFU. This animal did not seroconvert; become bacteremic or toxemic; or demonstrate increases above baseline in heart rate, respiration rate, or body temperature. End of study pathology examination showed minimal acute inflammation in the lung, which may or may not have been induced by exposure to the *B. anthracis* spores.

Based on the actual doses administered, the LD_50_ was determined to be 5.18 × 10^4^ CFU. As a point of comparison, the most often cited LD_50_ value for the rabbit reported by Zaucha et al. [18] LD_50_ was 1.05 × 10^5^, and the current study’s value was generally consistent with that value.

This is the first published study to report the pathology and histopathology for rabbits challenged with inhalation exposure to irradiated *B. anthracis* spores. These rabbits exhibited generally unremarkable gross findings and unremarkable findings in the microscopic evaluation of the lungs, with the exception of the presence of the minimal multi-nucleated giant cells that were described. However, many of the rabbits challenged with live spores that developed inhalation anthrax or remained infection-free also had multi-nucleated giant cells in the lungs at minimal to moderate levels. This finding did not correspond with challenge dose. Inhaled debris, including aggregations of spores, likely contributed to the development of multinucleated giant cells and granulomas.

Lawrence et al. reported the first comprehensive description of the physiological response of rabbits after high-dose nasal installation of *B. anthracis* spores [35]. This study complements the that work by reporting the physiologic response in the rabbit after low-dose aerosol challenge of *B. anthracis* spores and confirming similar physiological response for lethal inhalation anthrax regardless of high-or low-dose challenge levels. The data presented here also suggest a trend toward increases in measured heart and respiration parameters in the higher challenge dose groups relative to their pre-challenge baseline. Increases in heart and respiration rates above baseline measures were commonly associated with lethality and present immediately prior to death, with 90% (10/11) of animals exhibiting tachycardia and 100% (11/11) exhibiting an increased respiratory rate of 30 or more respiratory cycles per minute above baseline. However, almost all surviving animals experienced transient increases in heart rate and approximately half of the animals exhibited abnormal respiratory rates during the study duration without development of inhalation anthrax or lethality. All rabbits (11/11) that died on study had a significant increase in body temperature above baseline response. This was consistent with the findings of Dawson et al. [36] who used continuous monitoring to determine that a significant increase of three standard deviations in body temperature was correlative to death. Increased heterophils and increased liver enzymes have been associated with inhalational anthrax in the rabbit model [35]. While the highest dose groups had the highest levels of CRP, the majority of rabbits had detectable levels of CRP regardless of levels of the challenge dose, indicating that this parameter was not a good indicator of disease outcome.

While the above parameters show a physiological response to exposure over a certain threshold, they are not disease-specific and may be caused by a myriad of bacteria. The diagnostic indicators assayed here were bacteremia and toxemia. Overall, the data indicate that challenge doses of *B. anthracis* below the level sufficient to establish systemic infection do not produce observable physiological responses; however, doses that trigger a response result in death.

The data from our study indicate that a challenge dose of *B. anthracis* below an inhaled dose of 2.06 × 10^3^ CFU appears to be insufficient to establish systemic infection and produces only minimal physiological responses. Doses that promoted a systemic infection, as evidenced by physiological responses with bacteremia and toxemia measured at least once during the study, were more likely to be associated with death from inhalation anthrax.

The study also provides important data to inform evaluation of low-dose *B. anthracis* inhalation exposures in the human by providing low-dose exposure and response data from a human-relevant animal model. The determination of a low-dose inhalation exposure range in the rabbit for which *B. anthracis* systemic infection did not occur provides a preliminary indication that a threshold may be present in the dose–response relationship for *B. anthracis* systemic infection in other animal models and the human. While this will require further evaluation to confirm, it is an important finding for human health risk assessment and the selection of appropriate modeling approaches for dose–response relationships. The survival data were used in dose-dependent modeling [19,20] demonstrating the utility of the dose–response data for further analysis and risk assessment method development efforts. Further analysis of these data to increase their utility in human health risk assessment includes the development of interspecies extrapolation approaches to derive a human equivalent dose for response ranges in this study.

## Supplementary Materials

The following are available online at https://www.mdpi.com/2076-0817/9/6/461/s1, Table S1: Individual Challenge Doses, Table S2: Individual Hematology Data, Table S3: Individual Clinical Chemistry Data, Table S4: Summary of Individual Gross and Microscopic Observations.
