# Supplementary material for: Physiological Responses to a Single Low-Dose of Bacillus anthracis Spores in the Rabbit Model of Inhalational Anthrax
Source: Pathogens. 2020 Jun 11;9(6):461. doi: 10.3390/pathogens9060461 (PMC7350313; doi:10.3390/pathogens9060461)
Supplement: Supplementary file 1 [file pathogens-09-00461-s001.zip › Table S1. Individual Challenge Doses.docx]

Table S1. Group mean daily inhaled doses of aerosolized *B. anthracis* Ames strain spores. Mean group and individual animal doses, particle size and geometric standard deviation, mortality, and time to death data for the single inhaled low-dose challenge.

| **Group ID** | **Group Mean Daily Inhaled Dose, CFU (Standard Deviation)** | **Animal** | **Inhaled Dose (CFU/animal)** | **Mass Median Aerodynamic Diameter, (µm) (Geometric Standard Deviation)** | **Time to Death from Challenge Day (day)** |
| --- | --- | --- | --- | --- | --- |
| 1 | 2.00  (4.58 x 10^-1^)  Irradiated Spores | L23220 | 0 | 0.96 (1.33) | Survived |
|  |  | L23216* | 1.00 x 10^1^ |  | Survived |
|  |  | L23218 | 0 |  | Survived |
|  |  | L23223 | 0 |  | Survived |
|  |  | L23222 | 0 |  | Survived |
| 2 | 2.86 x 10^2^  (4.32 x 10^1^) | L23215 | 3.22 x 10^2^ | 0.82 (1.48) | Survived |
|  |  | L23206 | 2.98 x 10^2^ |  | Survived |
|  |  | L23210 | 2.18 x 10^2^ |  | Survived |
|  |  | L23219 | 3.21 x 10^2^ |  | Survived |
|  |  | L23211 | 2.73 x 10^2^ |  | Survived |
| 3 | 2.06 x 10^3^  (3.42 x 10^2^) | L23217 | 1.48 x 10^3^ | 0.92 (1.57) | Survived |
|  |  | L23230 | 2.02 x 10^3^ |  | Survived |
|  |  | L23228 | 2.23 x 10^3^ |  | Survived |
|  |  | L23227 | 2.32 x 10^3^ |  | Survived |
|  |  | L23229 | 2.24 x 10^3^ |  | Survived |
| 4 | 2.54 x 10^4^  (5.21 x 10^3^) | L23235 | 1.76 x 10^4^ | 0.87 (1.59) | 11 |
|  |  | L23205 | 2.73 x 10^4^ |  | Survived |
|  |  | L23225 | 2.59 x 10^4^ |  | 4 |
|  |  | L23231 | 2.41 x 10^4^ |  | Survived |
|  |  | L23207 | 3.19 x 10^4^ |  | Survived |
| 5 | 2.75 x 10^5^  (7.41 x 10^4^) | L23201 | 1.78 x 10^5^ | 1.12 (1.33) | 4 |
|  |  | L23234 | 2.96 x 10^5^ |  | 6 |
|  |  | L23212 | 3.29 x 10^5^ |  | Survived |
|  |  | L23200 | 2.19 x 10^5^ |  | 3 |
|  |  | L23214 | 3.54 x 10^5^ |  | 6 |
| 6 | 8.27 x 10^6^  (1.69 x 10^6^) | L23204 | 5.95 x 10^6^ | 1.12 (1.31) | 4 |
|  |  | L23203 | 8.86 x 10^6^ |  | 5 |
|  |  | L23213 | 7.29 x 10^6^ |  | 3 |
|  |  | L23221 | 8.88 x 10^6^ |  | 2 |
|  |  | L23232 | 1.04 x 10^7^ |  | 4 |

CFU – colony forming unit(s)
