# Supplementary material for: Physiological Responses to a Single Low-Dose of Bacillus anthracis Spores in the Rabbit Model of Inhalational Anthrax
Source: Pathogens. 2020 Jun 11;9(6):461. doi: 10.3390/pathogens9060461 (PMC7350313; doi:10.3390/pathogens9060461)
Supplement: Supplementary file 1 [file pathogens-09-00461-s001.zip › Table S4. Summary of individual gross and microscopic observations .docx]

Table S2. Summary of individual gross and microscopic observations

| **Dose Group** | **Animal Number/**  **Death Status** | **Gross Findings** | **Microscopic Findings** |
| --- | --- | --- | --- |
| 100 $\times$ LD_50_ (Irradiated) | L23220/FS |  | Lung: Unremarkable |
|  | L23216/FS |  | Lung: Unremarkable |
|  | L23218/FS |  | Lung: Multi-nucleated giant cells, minimal |
|  | L23223/FS |  | Lung: Unremarkable |
|  | L23222/FS |  | Lung: Multi-nucleated giant cells, minimal |
| 100 CFU | L23215/FS |  | Lung: Inflammation, suppurative, minimal  Lung: Multi-nucleated giant cells, minimal |
|  | L23206/FS |  | Lung: Inflammation, suppurative, minimal |
|  | L23210/FS |  | Lung: Unremarkable |
|  | L23219/FS |  | Lung: Inflammation, suppurative, minimal  Lung: Multi-nucleated giant cells, minimal |
|  | L23211/FS |  | Lung: Unremarkable |
| 1,000 CFU | L23217/FS |  | Lung: Inflammation, suppurative, moderate Lung: Multi-nucleated giant cells, moderate  Lung: Perivascular eosinophils, moderate |
|  | L23230/FS |  | Lung: Inflammation, suppurative, minimal |
|  | L23228/FS |  | Lung: Inflammation, suppurative, minimal  Lung: Multi-nucleated giant cells, minimal |
|  | L23227/FS |  | Lung: Bacteria (bacilli), minimal |
|  | L23229/FS |  | Lung: Inflammation, suppurative, minimal |
| 10,000  $\times$ LD_50_ | L23235/FD |  | Lung: Inflammation, suppurative, minimal |
|  | L2320)/FS |  | Lung: Inflammation, nonsuppurative, minimal  Lung: Perivascular eosinophils, minimal |
|  | L23225/FD | Cavity, abdominal: Fluid, red, ~30 mL  Cavity, thoracic: Fluid, red, ~15 mL | Lung: Inflammation, suppurative, mild  Lung: Bacteria (bacilli), moderate |
|  | L23231/FS | Skin: Crust(s), hindlimb, red, left hind limb,  20 mm x 20 mm | Lung: Inflammation, nonsuppurative, minimal  Skin: Necrosis, moderate |
|  | L23207/FS |  | Lung: Multi-nucleated giant cells, mild  Lung: Bacteria (bacilli), minimal  Lung: Inflammation, nonsuppurative, minimal |
| 100,000  $\times$ LD_50_ | L23201/FD | Cavity, thoracic: Fluid, red, ~15 mL | Lung: Inflammation, suppurative, mild  Lung: Multi-nucleated giant cells, mild  Lung: Bacteria (bacilli), mild |
|  | L23234/FD | Cavity, thoracic: Fluid, red, ~10 mL  Skin: Crust(s), hindlimb, dark, left hindlimb, 30 mm $\times$ 5 mm  Skin: Crust(s), dark, back, 5 mm $\times$ 5 mm  Skin: Fluid, abdominal, clear, ventral abdomen, ~8 mL  Thymus: Fluid, clear, ~8 mL | Lung: Inflammation, suppurative, moderate  Lung: Multi-nucleated giant cells, mild  Lung: Bacteria (bacilli), moderate  Skin: Necrosis, mild  Skin: Bacteria (bacilli), moderate  Skin: Inflammation, suppurative, mild  Skin: Hemorrhage, mild  Thymus: Atrophy, lymphoid, moderate  Thymus: Edema, mild  Thymus: Bacteria, mild |
|  | L23212/FS |  | Lung: Inflammation, suppurative, minimal |

| **Group Number** | **Animal Number/**  **Death Status** | **Gross Findings** | **Microscopic Findings** |
| --- | --- | --- | --- |
| 100,000  $\times$ LD_50_ | L23200/FD | Cavity, thoracic: Fluid, red, ~20 mL | Lung: Inflammation, suppurative, minimal  Lung: Multi-nucleated giant cells, minimal |
|  | L23214/FD |  | Lung: Inflammation, suppurative, mild  Lung: Multi-nucleated giant cells, mild  Lung: Bacteria (bacilli), mild |
| 100 $\times$ LD_50_ | L23204/FD | Cavity, thoracic: Fluid, red, ~15 mL | Lung: Inflammation, suppurative, mild  Lung: Multi-nucleated giant cells, mild  Lung: Bacteria (bacilli), mild |
|  | L23203/FD |  | Lung: Inflammation, suppurative, mild  Lung: Multi-nucleated giant cells, mild  Lung: Bacteria (bacilli), mild |
|  | L23232/FD | Brain: Discoloration(s), meninges, diffuse, red, affects all lobes  Lymph node, bronchial: Enlarged, dark, 3x  Lymph node, mediastinal: Enlarged, dark, 3x | Brain: Inflammation, suppurative, moderate  Brain: Hemorrhage, mild  Brain: Bacteria, minimal  Lung: Inflammation, suppurative, moderate  Lung: Bacteria (bacilli), minimal  Lymph node, bronchial:  Fibrin, minimal  Hemorrhage, mild  Necrosis, lymphoid, mild  Bacteria (bacilli), minimal  Lymph node, mediastinal:  Fibrin, minimal  Histiocytosis, mild  Necrosis, lymphoid, mild  Necrosis, vascular, mild  Bacteria (bacilli), minimal  Thymus: Atrophy, lymphoid, mild  Thymus: Edema, mild |
|  | L23221/FD | Cavity, abdominal: Fluid, red, ~60 mL  Intestine, small: Fluid, jejunum, green, ~50 mL Abdomen was distended | Intestine, small:  Inflammation, suppurative, mild  Bacteria (bacilli), mild  Lung: Inflammation, suppurative, mild  Lung: Bacteria (bacilli), moderate |
|  | L23213/FD | Lymph node, mediastinal: Enlarged, dark, 3x | Lung: Inflammation, suppurative, mild  Lung: Bacteria (bacilli), minimal  Lymph node, mediastinal:  Fibrin, moderate  Hemorrhage, moderate  Necrosis, lymphoid, marked  Bacteria (bacilli), marked |

FD = Found dead

FS = Final phase sacrifice
